# Thinning Approximation for Calculating Two-Dimensional Scattering Patterns in Dissipative Particle Dynamics Simulations under Shear Flow

**DOI:** 10.3390/polym10111224

**Published:** 2018-11-03

**Authors:** Katsumi Hagita, Takahiro Murashima, Nobuyuki Iwaoka

**Affiliations:** 1 Department of Applied Physics, National Defense Academy, Yokosuka 239-8686, Japan; 2 Department of Physics, Tohoku University, Sendai 980-8578, Japan; murasima@cmpt.phys.tohoku.ac.jp; 3 Department of Creative Engineering, Tsuruoka College, National Institute of Technology, Yamagata 997-8511, Japan; niwaoka@tsuruoka-nct.ac.jp

**Keywords:** dissipative particle dynamics (DPD) simulations, two-dimensional scattering patterns (2DSPs), thinning approximation (TA), shear deformation

## Abstract

Modifications to improve thinning approximation (TA) were considered in order to calculate two-dimensional scattering patterns (2DSPs) for dissipative particle dynamics (DPD) simulations of polymer melts under a shear flow. We proposed multipoint TA and adaptive TA because the bond lengths in DPD chains vary widely when compared to those in Kremer–Grest (KG) chains, and the effectiveness of these two types of TA for the two major DPD parameter sets were investigated. In this paper, we report our findings on the original DPD model with soft bonds and that with rigid bonds. Based on the behavior of the 2DSPs and the distribution of orientations of the bond vectors, two spot patterns originating from the oriented chain correlations were observed when distinct distributions of the highly oriented bond vectors in the shear direction were obtained. For multipoint TA, we concluded that at least two additional midpoints ($n_{\mathrm{mid}}\geq 2)$ are required to clearly observe the two spot patterns. For adaptive TA, a dividing distance of $l_{\mathrm{ATA}}\leq 0.4$ is sufficient for clear observation, which is consistent with the requirement of $n_{\mathrm{mid}}\geq 2$ for multipoint TA.

## 1. Introduction

Dissipative particle dynamics (DPD) [1,2,3] simulations are widely performed in order to study the dynamic and rheological properties of simple and complex fluids such as polymer melts and solutions [4,5,6,7,8], block copolymers [9,10,11,12,13,14,15], lipids [16,17,18,19], and colloidal suspensions [20,21,22]. One advantage of the DPD method is that it allows longer time and length scales than conventional molecular dynamics (MD) simulations. The particles in DPD simulations are more coarse-grained than those of standard bead–spring models such as the Kremer–Grest (KG) model [23]. In terms of computing costs, those for DPD simulations are reasonable for the study of polymeric fluids. In general, we believe that the orientation distributions of polymers in polymeric fluids depend on the local velocities of the fluids. The orientations of polymer chains are generally investigated by analyzing two-dimensional scattering patterns (2DSPs) obtained in wide-angle X-ray scattering (WAXS) experiments [24]. Since polymers in a melt have a disordered structure, their 2DSPs show a foggy ring without crystalline diffraction; this ring pattern is called an amorphous halo [25]. When polymer chains are oriented in a certain direction, the amorphous halo splits into two foggy spots in the direction perpendicular to the orientation of the polymer chains [24,26,27]. In order to investigate the molecular orientation of polymer chains, researchers have examined 2DSPs of amorphous and semicrystalline polymers under uniaxial deformation [27], liquid crystalline polymers under shear flows [28], polyethylene and poly(vinylidene fluoride) fibers under shear flows [29], polyisoprene rubber and natural rubber under uniaxial deformation [30], and isotactic polypropylene under shear flows [31].

For block copolymer systems, 2DSPs were extensively observed to study structural changes due to shear or uniaxial deformations [32,33,34,35,36,37,38,39,40,41,42]. For example, Betes et al. [32] reported the dependence of the order–disorder transition in poly(ethylene-propylene)–poly(ethylethylene) (PEP–PEE), a diblock copolymer, on the shear rate; two-dimensional contour plots of the two phases show changes corresponding to those in scattering patterns. Okamoto, Saijo, and Hashimoto performed real-time SAXS observations of lamella-forming block copolymers under large-amplitude oscillatory shear deformation [33]. In a notable study, Matsushita et al. examined anisotropic 2DSPs of the alternating lamellar structures of ABA triblock copolymers without shear or uniaxial deformations [43]. Vigild et al. [34] reported changes in 2DSPs of poly(ethylene-propylene)–poly(dimethylsiloxane) (PEP–PDMS), a diblock copolymer, to study the correspondence between their rheological and structural properties. Reynders et al. [35,36] reported changes in 2DSPs of a micellar network of polystyrene–poly(ethylene-propylene)–polystyrene (PS–PEP–PS), a triblock copolymer, in oil under elongation. Krishnamoorti et al. conducted neutron scattering experiments to examine 2DSPs during a cylinder-to-sphere order–order transition in block copolymers [37]. Sakurai and co-workers examined 2DSPs of strain-induced deformation of glassy spherical micro-domains in elastomeric triblock copolymers [38,39,40]; they also measured the stress as a function of strain in these systems. Mao et al. and McCready and Burghardt reported changes in 2DSPs corresponding to deformation of re-oriented micro-domains in a hexagonally ordered styrene–ethylene–butylene–styrene (SEBS) triblock copolymer under uniaxial extensional flow [41,42].

**Figure 1:**
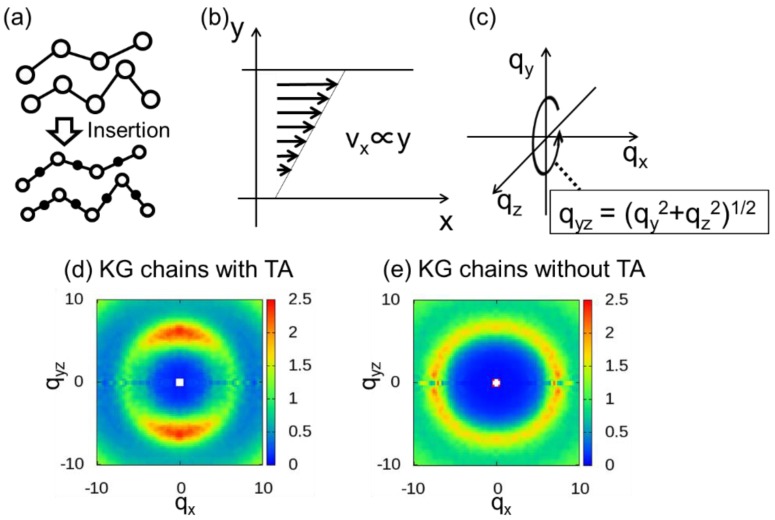
Schematic illustrations of (**a**) the thinning approximation (TA) process; (**b**) the velocity profile obtained at a constant shear rate; and (**c**) the circular average; two-dimensional scattering patterns (2DSPs) of Kremer–Grest (KG) chains under shear flow (**d**) with TA and (**e**) without TA. The presented 2DSPs were partially reprinted from an earlier paper, Hagita, K.; Murashima, T.; Takano, H.; Kawakatsu, T. *J. Phys, Soc. Jpn*. **2017**, 86, 124803. [44], with the permission.

Although many studies based on the DPD method and the KG model were published, very few were conducted on 2DPSs. One of the reasons was that two spots originating from bond orientation cannot be reproduced. Recently, one of this work’s authors, Hagita, and co-workers developed the method of thinning approximation (TA) to estimate 2DSPs of KG chains under shear [44]. TA was introduced to deduce information on the bond orientation of chains. Although the coarse-grain (CG) model usually offers a picture of the chains, the chain picture is lost when the model is used for dense polymer melts because the density field of particles of the polymer chains is almost homogeneous. It was confirmed that TA is effective for calculating 2D WAXS patterns of dense polymer melts under a rapid shear flow. The approximation consists of (1) midpoint insertion and (2) determination of a circular average to improve the statistics. The circular average is based on the assumption of rotational symmetry around the $q_{x}$ axis of the scattering vector *q*, where the *x* axis is the elongation axis. The TA process is illustrated in Figure 1. Figure 1a shows an example of polymer chains before and after TA, where the filled circles represent inserted pseudo-particles. Figure 1b shows the velocity profile of shear deformation at a constant shear rate. Figure 1c shows the circular averaging based on the assumption of rotational symmetry around the $q_{x}$ axis, and $q_{yz}$ is defined as $q_{yz}={\left(q_{y}^{2}+q_{z}^{2}\right)}^{1/2}$.

Although TA with midpoints was successful for KG chains, we found that it did not work for DPD chains because the fluctuations in bond length were larger in DPD chains than those in KG chains. To resolve this problem, we inserted multiple points in the bonds, and we expected the number of points required for a certain bond to depend on its bond length. In this paper, we report the cases with a fixed number of inserted points and cases with an adaptive (i.e., variable) number of inserted points. Hereafter, we refer to the first case as multipoint TA and the second case as adaptive TA (ATA).

In our study, we examined 2DSPs of dense polymer melts under a rapid shear flow to confirm the effectiveness of multipoint TA and ATA for DPD simulations. To simulate shear flows, the SLLOD (transposed Doll’s tensor) algorithm [45] was used with the Lagrangian rhomboid boundary conditions [46,47], which are equivalent to the Lee–Edwards boundary conditions [48].

In Section 2, we explain methods of the DPD simulations and the calculation of 2DSPs with multipoint and adaptive TA. In Section 3, we examine the proposed improvements of TA for DPD simulations. A summary of the results and our conclusions are presented in Section 4.

## 2. Methods and Model

### 2.1. DPD Simulations

Groot and Warren [3,9] proposed the DPD method for polymeric systems. A DPD polymer is modeled as soft-core particles (segments) connected by springs. In the DPD model, the soft-core potential represents a collection of monomers, and the dynamics of the soft-core segments are solved. In this study, we considered melts of *M* polymers with monodisperse chain length (*N* segments). In this study, we focused on the monodisperse chain length that is characterized by the Weisenberg number. For the polymers with polydisperse chain length, the spot for high shear rate is expected to be unclear because the short chains will quickly relax and will not be oriented. Research on this type of polymers is currently in progress.

In the DPD [1,2,3] method, the time evolution of the velocity, $v_{i}$, of the *i*th segment is governed by the following equation of motion:

(1) $$m\frac{dv_{i}}{dt}=\sum_{j\neq i}F_{i,j},$$

where *m* denotes the mass of a segment of the homopolymer, $F_{i,j}$ is the force from the *j*th segment acting on the *i*th segment. Here, $v_{i}$ is also given by the time-derivative of the position, $r_{i}$: $dr_{i}/dt=v_{i}$,. The force is given by the sum of the non-bonded conservative force, $F_{i,j}^{C}$; dissipative force, $F_{i,j}^{D}$; random force, $F_{i,j}^{R}$; and bonded force: $F_{i,j}^{B}$; $F_{i,j}=F_{i,j}^{C}+F_{i,j}^{D}+F_{i,j}^{R}+F_{i,j}^{B}$.

The non-bonded conservative force is a soft repulsive force, and it is represented by:

(2) $$F_{i,j}^{C}\left(r_{i,j}\right)=\{\begin{array}{c} a_{i,j}\left(1-\frac{r_{i,j}}{r_{c}}\right){\hat{r}}_{i,j}\;\mathrm{for}\;r<r_{c}\\ 0\;\mathrm{for}\;r\geq r_{c} \end{array},$$

where $a_{i,j}$ and $r_{c}$ are the force constant and cutoff distance, respectively; $r_{i,j}=\left|r_{i}-r_{j}\right|$; and ${\hat{r}}_{i,j}=\left(r_{i}-r_{j}\right)/r_{i,j}$. Generally, the dissipative and random forces are given by

(3) $$F_{i,j}^{D}\left(r_{i,j}\right)=-\gamma {\left(1-\frac{r_{i,j}}{r_{c}}\right)}^{2}\left({\hat{r}}_{i,j}\cdot v_{i,j}\right){\hat{r}}_{i,j},$$

(4) $$F_{i,j}^{R}\left(r_{i,j}\right)=\sigma \theta_{i,j}\left(1-\frac{r_{i,j}}{r_{c}}\right){\hat{r}}_{i,j},$$

where $v_{i,j}=v_{i}-v_{j}$, and Δt is the time step. The randomly fluctuating variables, $\theta_{i,j}$, obey Gaussian statistics, where $\langle \theta_{i,j}\left(t\right)\rangle =0$ and $\langle \theta_{i,j}\left(t\right)\theta_{k,l}\left(t^{\prime }\right)\rangle =\left(\delta_{i,k}\delta_{j,l}+\delta_{i,l}\delta_{j,k}\right)\delta \left(t-t^{\prime }\right)$. The noise amplitude, *σ*, is related to the dissipation factor, *γ*, as follows:

(5) $$\sigma^{2}=2\gamma k_{B}T,$$

where $k_{B}$ and T denote the Boltzmann constant and temperature, respectively. The canonical ensemble in Equation (5) was confirmed by Espanõl and Warren to be satisfactorily represented by the DPD model [2]. Thus, σ is called the DPD thermostat parameter. The force in a harmonic spring connecting two bonded segments in a polymer chain [3,9] can be described by

(6) $$F_{i,j}^{B}\left(r_{i,j}\right)=-K\left(r_{i,j}-r_{0}\right){\hat{r}}_{i,j},$$

where K and $r_{0}$ are the spring constant and equilibrium bond length, respectively. In this study, we used the reduced units of mass (*m*), length (*r*_c_), energy (*k*_B_*T*), and time (*τ*), i.e., m=1, $r_{c}=1$, $k_{B}T=1$, and $\tau =r_{c}\sqrt{m/k_{B}T}$, respectively, and the time step was set to Δt=0.01. In the present paper, we used the Large-scale Atomic/Molecular Massively Parallel Simulator (LAMMPS) [49] for all of the simulations.

We examined two types of DPD polymer with different bond springs. The first type of DPD polymer has “soft” bonds, whereas the second type has “rigid” bonds. Table 1 lists the DPD parameters used for these two types of polymer. In the original work of Groot and Warren [3,9], the soft bond corresponds to the optimized DPD parameters. Since then, the soft bond has been widely employed in DPD simulations [10,11,12,13,14,15] to study the morphology of block copolymer materials. The rigid bond has been used to reduce bond crossings [50,51,52,53,54,55,56,57,58]. For efficient reduction of the bond crossings, segmental repulsive potential models have been widely studied [52,53,54,55,56,57,58]. Note that the segmental repulsive potential is not taken into account in this work because our focus is the steady states of polymer melts under a rapid shear flow, where the polymer chains are highly oriented to the flow direction.

### 2.2. Two-Dimensional Scattering Patterns

The scattering intensity I(q) is a function of the density field ρ(R(i,j,k)) in the observed system, where R(i,j,k) is the position of the grid point (i,j,k), which is the index of a three-dimensional (3D) regular mesh grid. We can obtain ρ(R(i,j,k)) from the position vector, r(n)  (where n=1,⋯,MN), which denotes the position of the *n*th segment of the polymer. Using  ρ(R(i,j,k)), I(q) can be calculated as follows:

(7) $$\frac{I\left(q\right)}{I\left(0\right)}=\frac{{\left|\sum_{i,j,k}\;\rho \left(R\left(i,j,k\right)\right)\;\exp\left(-iq\cdot R\left(i,j,k\right)\right)\right|}^{2}}{{\left|\sum_{i,j,k}\;\rho \left(R\left(i,j,k\right)\right)\;\right|}^{2}}.$$

For efficient computing of the numerator in Equation (7), we often use 3D fast Fourier transformation (3D-FFT), where:

(8) $$\mathrm{FT}\left[\rho \left(R\left(i,j,k\right)\right)\right]=\underset{i,j,k}{\sum }\rho \left(R\left(i,j,k\right)\right)\;\exp\left(-iq\cdot R\left(i,j,k\right)\right).$$

When the scattering elements are points, I(0) is given by the product of squares of the scattering factors of the elements and the number of elements. In the present work, the scattering factors were set to unity so that $I\left(0\right)=MN=\left|\underset{i,j,k}{\sum }\rho \left(R\left(i,j,k\right)\right)\right|$. For the insertion of additional midpoints, we must keep the total scattering factors the same; therefore, we scaled the scattering factor of each scattering site, which consists of segments of DPD chains and the additional midpoints. The intensity of each point on a 2DSP, $I\left(q_{x},q_{y}\right)$, is defined on the plane of I(q), with $q_{z}=0$. To calculate the X-ray and neutron scattering patterns corresponding to the all-atomistic molecular dynamics (AAMD) model, the density field ρ(R(i,j,k)) can be estimated from the atomic position and atomic weight. For the X-ray and neutron scattering patterns, the weight for ρ(R(i,j,k)) corresponds to the atomic number and the scattering length density, respectively, of the atoms. In general, the chain picture is required to calculate the wide-angle scattering intensity, which is related to the orientation distribution of bonds. To overcome this problem, we propose using TA to calculate the 2D WAXS patterns of dense polymer melts with KG chains under a rapid shear flow. Figure 1a shows an illustration of the TA process based on the insertion of particles in the middle of bonds. In our previous work [44], the total volume is assumed to be conserved from the viewpoint of the scattering length density. For the fine-graining of dense KG chains with extra monomers placed in the middle of bonds, the effective volume of each monomer shrinks by 1/2. Based on Equation (7), I(q) is independent of the scaling factor, *α*, for *ρ*. Here, *α* is proportional to the volume of one monomer and corresponds to the scattering length density for X-ray scattering. Owing to the conservation of the total volume, *α* = 1/2 because the number of monomers becomes 2*N* after the TA. Thus, we can assume that the diameter of the polymer chains decreases to 2^−1/3^. For the DPD chains, the concept of TA is effective. Owing to the large distribution of bond lengths when compared to those of KG chains, we assumed multiple (a fixed number and an adaptive number) midpoints, $n_{\mathrm{mid}}$. Here, the TA for KG chains [44] corresponds to the cases with $n_{\mathrm{mid}}$ = 1, and the scaling factor *α* of the effective volume of each scattering site used to calculate ρ(R(i,j,k)) is given by $\alpha =1/\left(n_{\mathrm{mid}}+1\right)=1/2$. Thus, we used $\alpha =1/\left(n_{\mathrm{mid}}+1\right)$ for multipoint TA. For adaptive TA, the scaling factor, α=1/(n+1), for each bond was set based on the number of divisions, n, obtained for each bond based on the dividing distance, $l_{\mathrm{ATA}}$. When multiple midpoints (i.e., multiple scattering sites) were placed on a bond, we used a fine mesh for ρ(R(i,j,k)) in the 3D-FFT.

For the anisotropic case along the *x* axis, in order to obtain better 2D scattering patterns, we calculated the average $I\left(q_{x},q_{y}\right)$ for each 2DSP, which is given by the circular average of I(q) on the $q_{y}-q_{z}$ plane, as shown in Figure 1c:

(9) $$I\left(q_{x},q_{yz}\right)=\frac{\sum_{\left\{\left(q_{x},q_{yz}\right)|q_{y}^{2}+q_{z}^{2}=q_{yz}^{2}\right\}}I\left(q\right)}{\sum_{\left\{\left(q_{x},q_{yz}\right)|q_{y}^{2}+q_{z}^{2}=q_{yz}^{2}\right\}}1},$$

where $q_{yz}={\left(q_{y}^{2}+q_{z}^{2}\right)}^{1/2}$.

As shown in our previous investigation [44] on KG chains, the observed anisotropy of the 2DSP on the *y* axis is as small as the random noise. Thus, the circular average with rotational symmetry on the $q_{y}-q_{z}$ plane seems to be reasonable even though it ignores the anisotropy on the *y* axis. Therefore, we assumed rotational symmetry of the system to improve the statistical accuracy of the 2DSP. Note that the number of grids, *n*, is given by $\;L_{\mathrm{pbc}}/\Delta x$ in the present work. Here, the length of each side of the box with periodic boundary conditions (PBCs) is $L_{\mathrm{pbc},x}=\;L_{\mathrm{pbc},y}=\;L_{\mathrm{pbc},z}=\;L_{\mathrm{pbc}}$ and the mesh size in real space is Δx=Δy=Δz. In this case, the mesh size for the 3D-FFT is given by $\Delta q_{x}=\Delta q_{y}=\Delta q_{z}=\;2\pi /L_{\mathrm{pbc}}$. For the circular averaging, the mesh size for the 2D WAXS pattern is given by $\Delta q_{∥}=\Delta q_{x}$ and $\Delta q_{⊥}=\Delta q_{y}=\Delta q_{z}$.

## 3. Results

To improve TA for DPD simulations, we performed DPD simulations of *M* (= 120) homo-polymer chains with *N* (= 100) beads per chain. We began by studying the dependence of the orientation of bond vectors on the Weisenberg number in DPD simulations under shear. Based on the evaluated Weisenberg-number dependence, multipoint TA and adaptive TA were examined for DPD simulations with rigid and soft bonds.

### 3.1. Weisenberg-Number Dependence of Orientation of Bond Vectors in DPD Simulations under Shear

**Figure 2:**
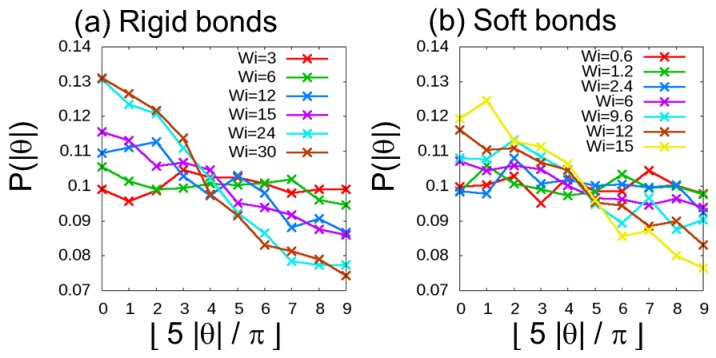
Probability, P(|θ|), versus ⎣5|θ|/π⎦ for dense polymer melts with (**a**) rigid and (**b**) soft bonds. Here, ⎣X⎦ is the floor function of X.

In rheology [60], the orientation of the bond vector of a polymer chain in melts under shear can be characterized by the Weisenberg number, *Wi*, which is defined as the product of the longest relaxation time, $\tau_{1}$, and the shear rate, $\dot{\gamma }$: $Wi=\tau_{1}\dot{\gamma }$. For the excluded-volume chain system that prohibits bond crossings, orientation is believed to take place when *Wi* > 1. For systems allowing bond crossings in DPD simulations, the threshold value of *Wi* is considered to change, so it is necessary to directly investigate this value. For various shear rates, $\dot{\gamma }$, the probability, P(|θ|), of the bond orientation parameter, |θ|, was estimated, where θ is given by $\cos\theta =x/\sqrt{x^{2}+y^{2}}$. Before simulations under shear were carried out, we estimated the longest Rouse relaxation time, $\tau_{1}$, of a chain in the melt for the calculation of Wi. For the case with *N* = 100 beads per chain, $\tau_{1}$ is about 1200τ and 3000τ (where *τ* is the reduced unit of time, $\tau =r_{c}\sqrt{m/k_{B}T})\;$ for the simulations of dense polymer melts with rigid and soft bonds, respectively. Figure 2 shows histograms of the probabilities P(|θ|) versus ⎣5|θ|/π⎦ for rigid and soft bonds, where ⎣X⎦ is the floor function of X. In our previous study [44] on the KG model, when the orientation distribution P(|θ|) with ⎣5|θ|/π=0⎦ increased by about 10% compared to that at the stationary state ($\dot{\gamma }=0$), two spot patterns originating from shear deformation were observed. Therefore, in the present study, we investigated Wi of the DPD simulations in which P(|θ|) with ⎣5|θ|/π=0⎦ increases by about 10%. We found that P(|θ|) with ⎣5|θ|/π=0⎦ increases by more than ~10% when the value of Wi is larger than 12 for the case with rigid bonds and when Wi is larger than 6 for the case with soft bonds. In this study, we examined the cases with Wi=6, 12, and 30 for rigid bonds and with Wi=6 and 12 for soft bonds. 

### 3.2. Thinning Approximation with Fixed Number of Additional Midpoints

#### 3.2.1. Rigid Bonds

**Figure 3:**
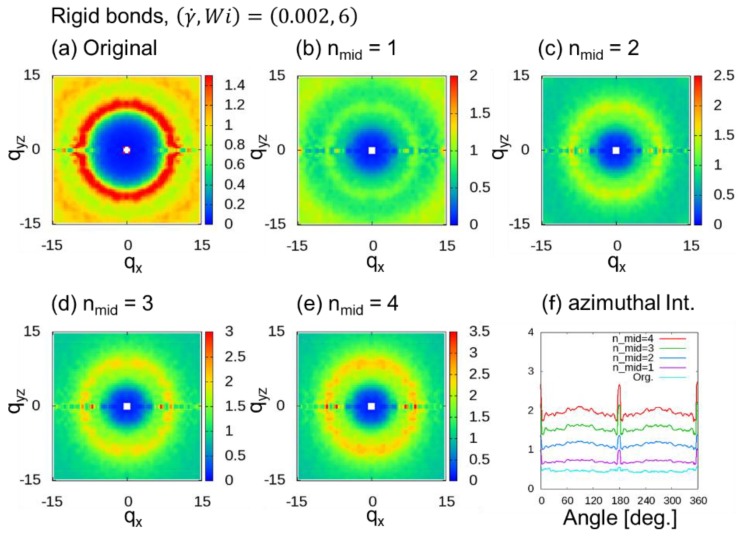
Two-dimensional scattering patterns (2DSPs) of DPD simulations of a dense polymer melt with rigid bonds for the cases with $\left(\dot{\gamma },Wi\right)=\left(0.002,\;6\right)$. The 2DSPs were calculated with and without thinning approximation (TA): (**a**) Original method; (**b**–**e**) $n_{\mathrm{mid}}$ additional points, where $n_{\mathrm{mid}}=1$ to 4; (**f**) azimuthal intensities averaged over from q = 6.0 to 8.0 with 0.1 steps.

**Figure 4:**
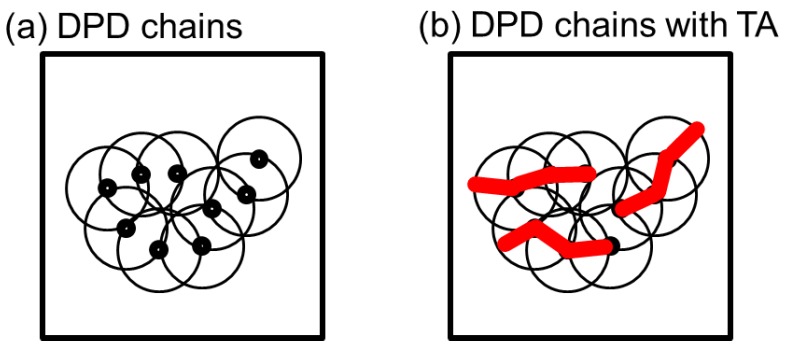
Schematic illustrations of (**a**) DPD chains and (**b**) additional midpoints for thinning approximation of a dense polymer melt. The circles denote the overlapping DPD particles. The red bold lines in (**b**) denote the series of additional midpoints. Based on the approximation, a clear picture of the chains was obtained.

We evaluated the behavior of 2DSPs in DPD simulations of a dense polymer melt with rigid chains under shear flow, where $\left(\dot{\gamma },Wi\right)=\left(0.002,\;6\right)$, (0.004, 12), and (0.01, 30) for $n_{\mathrm{mid}}$ (= 1 to 4) additional midpoints. Figure 3 shows 2DSPs for the cases with $\left(\dot{\gamma },Wi\right)=\left(0.002,\;6\right).$ For the cases with $\left(\dot{\gamma },Wi\right)=\left(0.002,\;6\right)$, since notable bond orientation is not observed in Figure 2, it is natural that two spot patterns originating from the bond orientation in the 2DSPs cannot be seen when we expect to see a ring pattern. As we increased $n_{\mathrm{mid}}$, we the found that the ring pattern becomes clearer for larger $n_{\mathrm{mid}}$. Note that this ring pattern originates from the correlation between the chains. As shown in Figure 4, the ring pattern is visible because the insertion of midpoints resulted in a clearer image of the chain.

**Figure 5:**
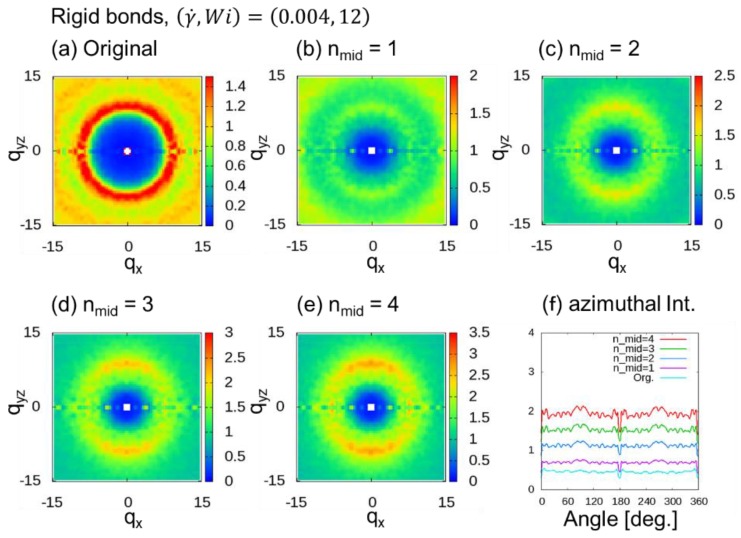
Two-dimensional scattering patterns (2DSPs) of DPD simulations of a dense polymer melt with rigid bonds for the cases with $\left(\dot{\gamma },Wi\right)=\left(0.004,\;12\right)$. The 2DSPs were calculated with and without thinning approximation (TA): (**a**) Original method; (**b**–**e**) $n_{\mathrm{mid}}$ additional points, where $n_{\mathrm{mid}}=1$ to 4; (**f**) azimuthal intensities averaged over from q = 6.0 to 8.0 with 0.1 steps.

Figure 5 shows 2DSPs for the cases with $\left(\dot{\gamma },Wi\right)=\left(0.004,\;12\right).$ When $n_{\mathrm{mid}}\geq 2$, two spot patterns originating from the bond orientation were observed. As we increased $n_{\mathrm{mid}}$, we found that the two spot patterns become clearer for larger $n_{\mathrm{mid}}$.

**Figure 6:**
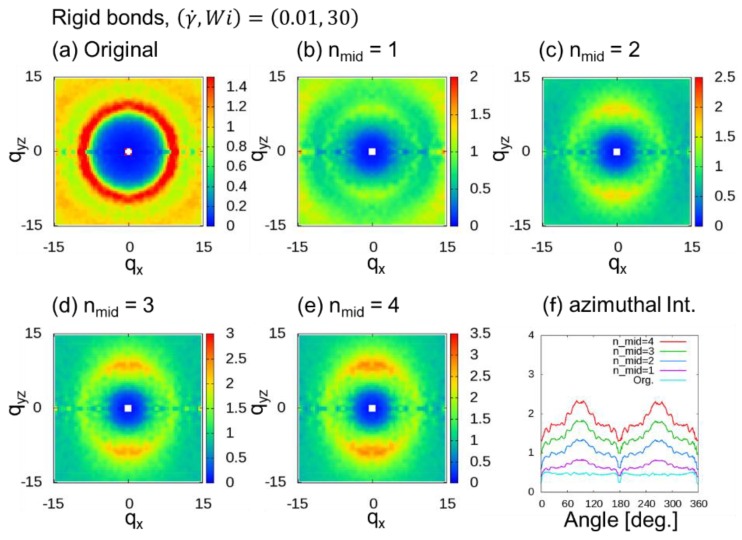
Two-dimensional scattering patterns (2DSPs) of DPD simulations of a dense polymer melt with rigid bonds for the cases with $\left(\dot{\gamma },Wi\right)=\left(0.01,\;30\right)$. The 2DSPs were calculated with and without thinning approximation (TA): (**a**) Original method; (**b**–**e**) $n_{\mathrm{mid}}$ additional points, where $n_{\mathrm{mid}}=1$ to 4; (**f)** azimuthal intensities averaged over from q = 6.0 to 8.0 with 0.1 steps.

Figure 6 shows 2DSPs for the cases with $\left(\dot{\gamma },Wi\right)=\left(0.01,\;30\right).$ When $n_{\mathrm{mid}}\geq 2$, two spot patterns originating from the bond orientation were observed, and they are clearer than the patterns shown in Figure 5. Even in this case, the two spot patterns were not observed when $n_{\mathrm{mid}}=1$. Therefore, for the case of DPD simulations with rigid bonds, at least two additional midpoints ($n_{\mathrm{mid}}\geq 2$) were necessary.

#### 3.2.2. Soft (Groot–Warren) Bonds

**Figure 7:**
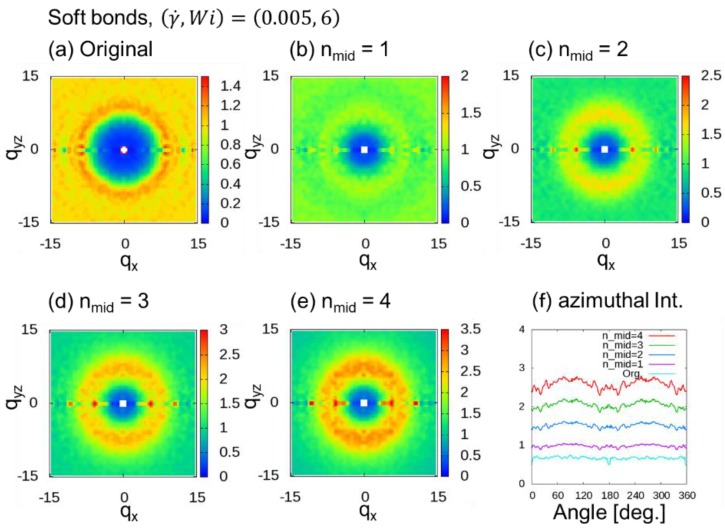
Two-dimensional scattering patterns (2DSPs) of DPD simulations of a dense polymer melt with soft bonds for the cases with $\left(\dot{\gamma },Wi\right)=\left(0.005,\;6\right)$. The 2DSPs were calculated with and without thinning approximation (TA): (**a**) Original method; (**b**–**e**) $n_{\mathrm{mid}}$ additional points, where $n_{\mathrm{mid}}=1$ to 4; (**f**) azimuthal intensities averaged over from q = 6.0 to 8.0 with 0.1 steps.

**Figure 8:**
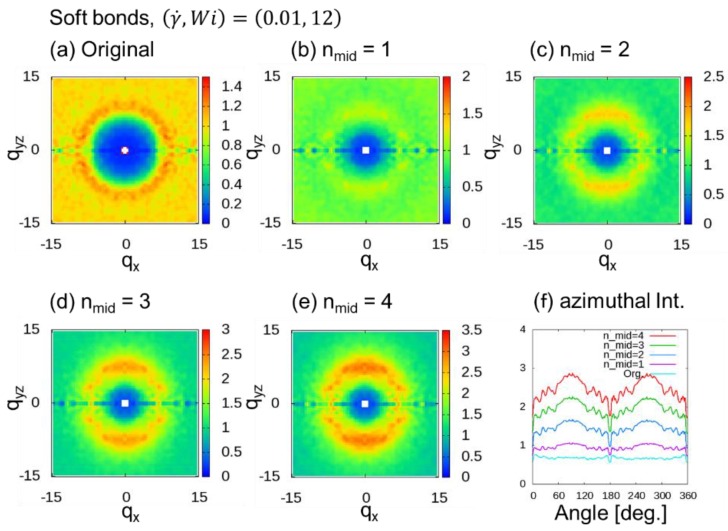
Two-dimensional scattering patterns (2DSPs) of DPD simulations of a dense polymer melt with the soft bonds for the cases with $\left(\dot{\gamma },Wi\right)=\left(0.01,\;12\right)$. The 2DSPs were calculated with and without thinning approximation (TA): (**a**) Original method; (**b**–**e**) $n_{\mathrm{mid}}$ additional points, where $n_{\mathrm{mid}}=1$ to 4; (**f**) azimuthal intensities averaged over from q = 6.0 to 8.0 with 0.1 steps.

We evaluated the behavior of the 2DSPs of DPD simulations of a dense polymer melt with soft chains under a shear flow, where $\left(\dot{\gamma },Wi\right)=\left(0.005,\;6\right)$ and (0.01, 12) for $n_{\mathrm{mid}}$ (= 1 to 4) additional midpoints. Figure 7 shows 2DSPs for the cases with $\left(\dot{\gamma },Wi\right)=\left(0.005,\;6\right).$ When $n_{\mathrm{mid}}\geq 2$, ring patterns originating from the chain correlations were observed. In this case, we also observed that the two spot patterns tend to be weak. Figure 8 shows 2DSPs for the cases with $\left(\dot{\gamma },Wi\right)=\left(0.01,\;12\right).$ When $n_{\mathrm{mid}}\geq 2$, two clear spot patterns originating from the bond orientation were observed. Therefore, in the case of the DPD simulations with soft bonds, at least two additional midpoints ($n_{\mathrm{mid}}\geq 2$) were necessary.

### 3.3. Adaptive Thinning Approximation (ATA)

**Figure 9:**
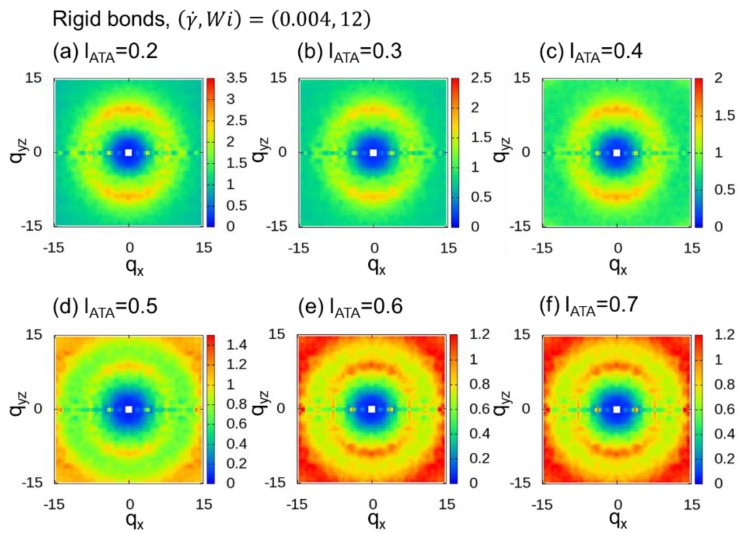
Two-dimensional scattering patterns (2DSPs) of DPD simulations of a dense polymer melt with rigid bonds for the cases with $\left(\dot{\gamma },Wi\right)=\left(0.004,\;12\right)$. The 2DSPs were calculated for $l_{\mathrm{ATA}}=0.2,0.3,\;0.4,\;0.5,\;0.6$, and 0.7.

**Figure 10:**
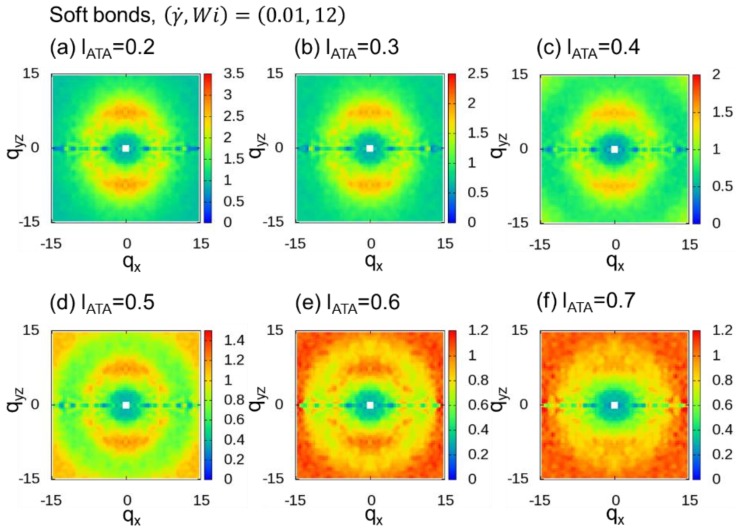
Two-dimensional scattering patterns (2DSPs) of DPD simulations of a dense polymer melt with soft bonds for the cases with $\left(\dot{\gamma },Wi\right)=\left(0.01,\;12\right)$. The 2DSPs were calculated for $l_{\mathrm{ATA}}=0.2,0.3,\;0.4,\;0.5,\;0.6$, and 0.7.

In THE DPD simulations, the fluctuations in bond length are much larger than those observed in coarse-grain molecular dynamics (CGMD) simulations of KG chains. In particular, the fluctuations were remarkable for DPD simulations of polymer melts with soft bonds, as shown in Figure 7 of the work of Iwaoka et al. [60]. We examined the average value and distribution of bond lengths in the polymer melt with rigid bonds and the polymer melt with soft bonds. The average, standard deviations, and squared average of bond length are 0.860, 0.066, and 0.862, respectively, for the rigid bond and 0.891, 0.291, and 0.938, respectively, for the soft bond. In addition to the method that includes a fixed number, $n_{\mathrm{mid}}$, of additional midpoints, another promising method includes a number of additional midpoints based on the dividing distance, $l_{\mathrm{ATA}}$. This method of determining the flexible *n*_mid_ is called adaptive thinning approximation (ATA), as noted in Section 1. We calculated 2DSPs with $l_{\mathrm{ATA}}=0.2,\;0.3,\;0.4,\;0.5,\;0.6$, and 0.7 for both cases with rigid (Figure 9) and soft (Figure 10) bonds. When $l_{\mathrm{ATA}}\leq 0.4$, two clear spot patterns were observed for both cases. When $l_{\mathrm{ATA}}\geq 0.5$, we can see the ring patterns originating from $l_{\mathrm{ATA}}$ in the range of $-15<q_{x}<15$ and $-15<q_{yz}<15$. Since the value obtained by dividing the average DPD bond length (0.860 and 0.891 for the rigid and soft bonds, respectively) by 0.4 is approximately 2, the value of $l_{\mathrm{ATA}}=0.4$ seems to be consistent with the corresponding observation of two spot patterns when $n_{\mathrm{mid}}\geq 2$, as reported in Section 3.1.

## 4. Summary and Conclusions

We considered thinning approximation for DPD simulations in order to calculate 2DSPs of dense polymer melts under a rapid shear flow with the SLLOD algorithm [45]. We investigated multipoint TA and adaptive TA as improvements because the bonds of the DPD chains are much softer and have larger length distributions than those of KG chains. The DPD simulations solve the dynamics of soft-core particles (segments), each of which represents a collection of monomers. A DPD chain was modeled as a set of such segments connected by springs. The two major sets of DPD parameters sets used—the soft and rigid bonds as presented in Table 1—were compiled from data reported in the literature. Unlike the original DPD model [3,9] with the soft bonds, rigid bonds with the segmental repulsive potentials [52] were introduced to reduce bond crossings. From the simulations of shear deformation, we found that distinct distributions of highly oriented bond vectors in the shear direction emerge when Wi≥12 and Wi≥6 for the cases with the rigid and soft bonds. The 2DSPs show that the two spot patterns originating from the oriented chain correlations appear when Wi≥12 for the simulations with rigid and soft bonds. For the simulations with Wi=6 and the soft bonds, two weak spot patterns were observed even though the ring patterns are dominant. For multipoint TA, two clear spot patterns were observed in the 2DSPs when $n_{\mathrm{mid}}\geq 2$ for both rigid and soft bonds. Thus, we conclude that at least additional midpoints ($n_{\mathrm{mid}}\geq 2)$ were necessary. We also confirmed the effectiveness of adaptive TA when $l_{\mathrm{ATA}}\leq 0.4$, which is consistent with corresponding observation of two spot patterns when $n_{\mathrm{mid}}\geq 2$ for multipoint TA.

Bond crossings are considered to have an important effect on the rheological and mechanical properties. In order to eliminate bond intersection in the equilibrium state compared to the rigid-bond model, one of the authors proposed multipoint segmental repulsive potentials (MP-SRP) [60]. By prohibiting the bond crossings, the orientation of the bond vector is considered to be relatively strong. The behavior in this case with MP-SRP is qualitatively considered to be the same as the result of the DPD simulation presented in this paper; a detailed comparison of the results will be carried out in the near future.

Furthermore, when considering the behavior of oriented polymer chains and their 2DSPs in the flow field for comparison with experimental results, generalization of the flow field in simulations under PBCs is required. Recently, Nicholson and Rutledge released uniaxial extensional flows (UEF) packages [61] for LAMMPS [49] in order to obtain infinitely succeeding uniaxial extensional flows (UEFs). One of the authors extended the UEF package for Langevin dynamics and DPD dynamics to develop the UEF-EX (Uniform Elongational Flow EXtension) package [62]. Calculation of 2DSPs for simulations using this package under the Kraynik–Reinelt boundary conditions [63] will also be discussed in forthcoming papers.

## Figures and Tables

**Table 1:** Dissipative particle dynamics (DPD) parameters of the polymers modeled by soft and rigid bonds. This table was reproduced from an earlier paper, Iwaoka, N.; Hagita, K.; Takano, H. *J. Chem. Phys.* 2018, 149, 114901 [59], with the permission of AIP Publishing.

Polymer Type	*a_ij_*	*K*	*r*_0_	*σ*
Soft bond [3,9,10,11,12,13,14,15]	25	4.0	0.0	3.0
Rigid bond [52,53,54,55,56,57,58]	60	450	0.85	3.0
